# Implementation of a Mobile DBT App and Its Impact on Suicidality in Transitional Age Youth with Borderline Personality Disorder: A Qualitative Study

**DOI:** 10.3390/ijerph19020701

**Published:** 2022-01-08

**Authors:** Tobias Schiffler, Radhika Seiler-Ramadas, Siniša Štefanac, Sandra Haider, Hanna M. Mües, Igor Grabovac

**Affiliations:** 1 Center for Public Health, Department of Social and Preventive Medicine, Medical University of Vienna, Kinderspitalgasse 15, 1090 Vienna, Austria; tobias.schiffler@meduniwien.ac.at (T.S.); sandra.a.haider@meduniwien.ac.at (S.H.); igor.grabovac@meduniwien.ac.at (I.G.); 2 Center for Medical Statistics, Informatics and Intelligent Systems, Institute for Outcomes Research, Medical University of Vienna, Spitalgasse 23, 1090 Vienna, Austria; sinisa.stefanac@meduniwien.ac.at; 3 Department of Clinical and Health Psychology, Faculty of Psychology, University of Vienna, Liebiggasse 5, 1010 Vienna, Austria; hanna.muees@univie.ac.at

**Keywords:** borderline personality disorder, dialectical behavior therapy, mobile health, mobile application, suicidal ideation, non-suicidal self-injury, transitional age youth

## Abstract

Contemporary performance and accessibility are features that enable mobile devices to be increasingly beneficial in the context of optimizing the treatment of psychiatric disorders. Smartphones have the potential to effectively support psychotherapeutic interventions among adolescents and young adults who require them. In the present study, the use and subjective influence of a smartphone app with content from dialectical behavior therapy (DBT) was investigated among transitional age youth (TAY) with borderline personality disorder, focusing on suicidality and non-suicidal self-injury (NSSI), in a natural setting. A longitudinal qualitative approach was used by means of individual semi-structured interviews, where participants were asked about their experiences and associated emotions before and after a testing period of 30 days. A total of 13 TAY with a diagnosed borderline personality disorder between the ages of 18 and 23 were included. Six overarching themes were identified through qualitative text analysis: (1) experiences with DBT skills, (2) phenomenon of self-harm, (3) feelings connected with self-harm, (4) dealing with disorder-specific symptoms, (5) prevention of self-harm, and (6) attitude toward skills apps. In general, the provision of an app with DBT content achieved a positive response among participants. Despite a small change in the perception of suicidality and NSSI, participants could imagine its benefits by integrating their use of the app as a supportive measure for personal psychotherapy sessions.

## 1. Introduction

Personality disorders are characterized by severe disturbances of the personality and dysfunctional behavioral tendencies without a biological or psychological basis [1]. In the case of emotionally unstable personality disorders, as defined by the International Statistical Classification of Diseases and Related Health Problems 10th Revision (ICD-10), there is a marked tendency for the individual to act out impulses without considering the accompanying consequences, often with emotional instability and low impulse control. This manifestation of personality disorder is separated into two subtypes: the impulsive type, and the borderline type. Characteristics of the borderline type include disturbances in one’s self-image, goals, and inner preferences, with chronic feelings of inner emptiness, making volatile social contacts, and having a tendency toward self-destructive behavior in the form of non-suicidal self-injury (NSSI) and suicide attempts [1,2]. Persons diagnosed with borderline personality disorder (BPD) commit an average of three suicide attempts over their lifetime [3]. Epidemiological research suggests a prevalence of BPD in the overall juvenile population of approximately 3% [4]. The clinical prevalence of the disorder ranges from 11% of adolescents in outpatient clinics to 78% of severely suicidal adolescents in emergency departments. However, it should be noted that there is a potential diagnostic bias, which does not rule out an equal frequency distribution [5]. NSSI mostly takes the form of superficial cuts to the wrists and arms with non-suicidal intent, but is based on the emotion-regulatory problems of the affected person. In this context, NSSI is an action which is executed in an addictive way to reduce painful internal states [6]. Apart from BPD, suicidal ideation is also a vast problem in the general global population of adolescents and young adults. Based on the life-threatening character and high prevalence of suicidal ideation and behaviors, they should be considered a high public health priority [7]. On a global basis, estimates for suicides that occurred in the age group of 10- to 29-year-olds exceeded 150,000 cases in 2019 [8]. Despite various efforts to reduce suicides through preventive initiatives over the past several years, they continue to be the fourth leading cause of death in people aged 15 to 29 years, after road injury, tuberculosis, and interpersonal violence. Suicide rates have increased dramatically in several countries and regions in recent years and show a high probability of a long-lasting upward trend due to the ongoing COVID-19 pandemic as well as economic recession [9,10,11,12].

One option for psychotherapeutic treatment of individuals with BPD is the concept of dialectical behavior therapy (DBT) developed by Marsha M. Linehan [13]. DBT is a comprehensively researched form of psychosocial treatment whose effectiveness in the care of individuals with BPD and associated problems has been previously verified as shown in a systematic review [14]. The majority of this research specifically reviewed BPD with suicidal behavior as the primary outcome [15]. DBT is a profound, multimodal treatment that supports chronically suicidal persons with BPD-specific deficits in emotional self-regulation and distress tolerance skills. This treatment is accomplished through a motivational model that aims to help affected individuals to overcome these deficits [16]. This model also assumes that personal and environmental factors often inhibit the use of innate behavioral skills present in persons with BPD. The importance of skills practice in DBT has already been established in empirical studies [17,18].

As a result of continuous development and improvement in accessibility to technological aids such as the internet and mobile applications (apps), studies were carried out to determine the effectiveness of their use in psychotherapeutic interventions. In particular, these technologies were examined with respect to their complementary and optimizing features when used in addition to personal therapy [19]. The use of smartphone-based interventions can have certain benefits in the therapeutic process. These include facilitating access to evidence-based treatment, increasing the effectiveness of psychotherapy, reducing stigma associated with psychotherapy, empowering users to explore content at their own pace, promoting user autonomy, and integrating flexible mental health interventions into everyday life without local or temporal constraints [20,21,22]. In terms of effectiveness, user willingness was identified as a key component in the successful implementation of a mobile app, regardless of the type of therapeutic intervention [23]. The integration of a mobile app into DBT suits the goals and techniques of psychotherapy for individuals with BPD [24]. However, the potential benefits of smartphone utilization must also be weighed against possible negative aspects, such as cyber-attacks and the illegal use of private data saved within the app [25]. Moreover, high frequency of mobile phone use among young adults is a potential risk factor for depressive symptomatology in both sexes and sleep disturbances in men [26]. A lower frequency use of skills from the DBT model was found to correlate with higher failure rates in therapy, while higher frequency use of these skills was associated with less NSSI. By means of integrating an app into the therapy process, appropriate therapeutic techniques can be made more accessible and the failure rate of individuals in treatment can be diminished [27].

This study focused on a group of transitional age youth (TAY), defined as a heterogeneous population between the ages of 15 and 26, namely individuals in their adolescence to early adulthood. Transitional age is characterized by emotional instability, self-focus, the absence of a sense of belonging, and sometimes a new sense of possibility and independence. Therefore, TAY find themselves in an intense period of self-exploration [28]. BPD gives rise to an intensification of these transitional age difficulties, which need to be counteracted by adequate measures in terms of harm reduction [29]. In order to properly address this issue, researchers developed an adaptation of DBT focusing on TAY [30]. The dialectical behavior therapy for adolescents (DBT-A) is specifically aimed at the age group of adolescents and young adults and focuses on the reduction of suicidal behaviors and NSSI that occur particularly in the context of BPD [31].

Therefore, the aim of the present study was to investigate whether using a mobile app with DBT content for 30 days influenced the perception of suicidal ideation and NSSI in TAY with BPD and how such an app could benefit this target group.

## 2. Materials and Methods

As the aim of this study was to map the perspectives of TAY with BPD through the use of a mobile app, a qualitative research approach was found to be most appropriate. This decision was especially based on the researchers’ intention of capturing the subjective experiences of participants during a period of app utilization, and exploring how its use influenced their daily life. The everyday experiences of participants as related to suicidal ideation and NSSI were of particular interest. In order to capture these as effectively as possible, the research process took place in the natural setting of participants.

### 2.1. Participants

Using convenience sampling, a total of 13 participants (12 women, 1 man) aged 18 to 23 years with diagnosed BPD were included in the study. The mean age was 20.1 years (SD 1.6). The sample consisted of former patients from a transitional psychiatric ward at the hospital of Hietzing in Vienna, Austria. Potential participants were selected according to the diagnosis in their clinical record and subsequently contacted by the principal investigator. In order to ensure a sufficient number of participants, snowball sampling and chain sampling were additionally used for recruitment.

The inclusion criteria were TAY between 18 and 25 years, sufficient understanding of the German language, a diagnosed BPD according to ICD-10, as well as experiencing NSSI or suicidal behavior in the past twelve months starting from the time of the first interview. Individuals were excluded if they experienced cognitive deficits, primary psychotic disorders, medically supervised drug withdrawal treatment, or a life-threatening eating disorder resulting in a BMI below 14.5. Eligibility criteria were assessed verbally in the course of the first contact with participants, and based on information provided by the hospital database. While language skills, self-harming behavior, state of drug withdrawal, and BMI were inquired about when potential participants were approached, data regarding age, diagnoses, and cognitive skills were available in the database. Participants were excluded if they needed inpatient treatment during the time of the study. Only participants who finished both interviews were included in the analysis.

### 2.2. Mobile App

The smartphone app used for the study was the mobile version of TalentLMS from the developer Epignosis. TalentLMS is a cloud-based platform for knowledge and learning management that allows knowledge transfer on a low-threshold basis by establishing easy access to specific content for users, enabling the individual creation of courses. TalentLMS provided an appropriate framework for a preferably simple implementation of the concept that was intended to be investigated, namely the offering of DBT-based content for TAY with BPD on a mobile device. In terms of the study, individual courses were created for each skills module within the German version of the DBT-A therapist manual of interactive skills training for adolescents with emotion regulation problems [30]. Since this manual consists of modules, it was possible to directly insert its content into the mobile app without further adaptations. For the implementation of this content, prior permission was obtained from the publisher holding the rights to the skills manual.

**Figure A1:**
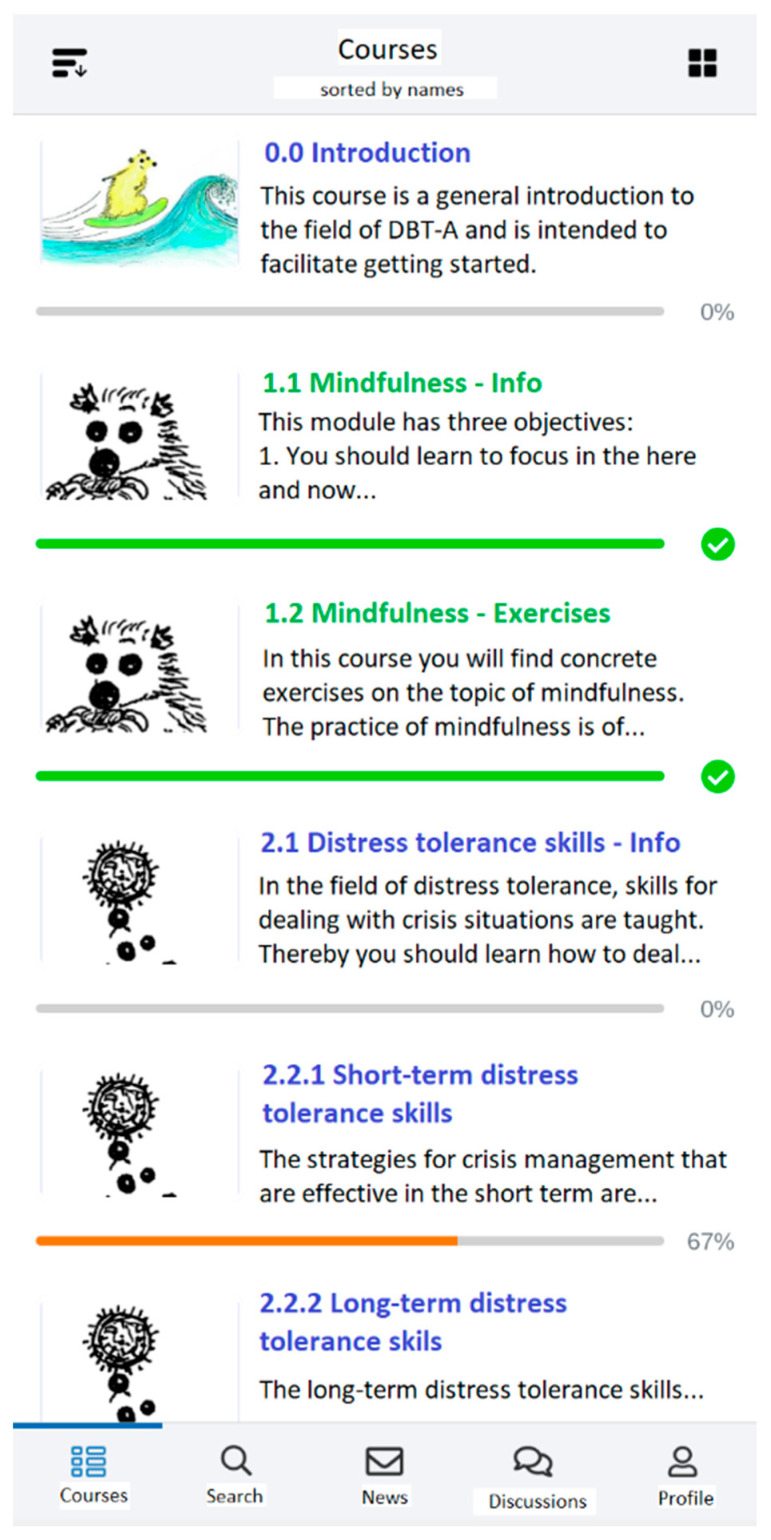
Overview of courses.

The app functioned purely as a source of information and did not offer the possibility to make entries. The initial stage involved browsing through the courses in a predefined order (Appendix A Figure A1). Thereafter, each individual course could be viewed separately according to individual preference. Users were then able to explore its content at their own discretion.

**Figure A2:**
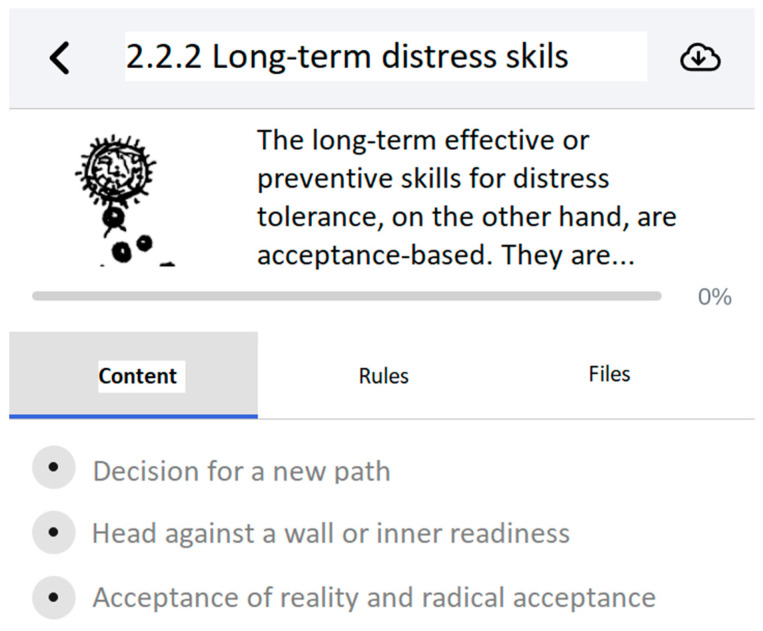
Tab “Content”.

**Figure A3:**
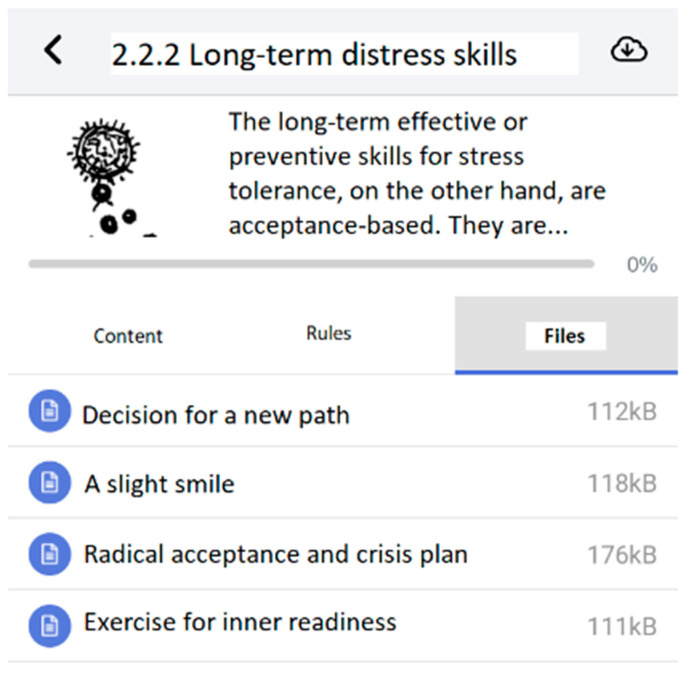
Tab “Files”.

Once a course was selected and opened, a new selection had to be made between the three tabs “Content”, “Rules”, and “Files”, although the “Rules” tab was not relevant. By default, the “Content” tab was selected (Appendix A Figure A2). This was also the main tab with information on specific skills. For each course, there was a short description of the content and goals of the respective module. Users could download worksheets for specific skills when they clicked onto the “Files” tab (Appendix A Figure A3).

In addition to the main tab “Courses”, there were also the “Search”, “News”, “Discussions”, and “Profile” tabs, which fulfilled various functions. Users were able to search for certain courses, course units, and course files. A course unit provided information for users about a basic theme of DBT-A, which comprised general theme descriptions and related exercises. Moreover, it was possible to post topics on a message board and to get directly in touch with the research team. Beyond that, it was possible to view one’s personal profile under the “Profile” tab. This allowed for checking one’s progress and scores in the respective courses for which users earned points and achievements upon completion.

The process of rolling out the app for participants was bound to the standards of TalentLMS, which provided its technical framework. After participants had submitted their informed consent, corresponding profiles were created in the course and the users were enrolled. Participants were informed about the exact test period and had access to the course content for 30 days from the beginning of May 2021. In addition to a verbal notification about the start of this test phase, participants were also informed by e-mail about the activation of the courses. Furthermore, they were instructed on how to get started with the system during an educational interview with the principal investigator, and open questions were clarified. The handling of the app was explained in a special in-app tutorial course where users were informed about the proper use of the application and the functions implemented in it. During the test period, users were not bound to any specifications or schemes regarding the use of the app.

### 2.3. Data Collection

**Figure A4:**
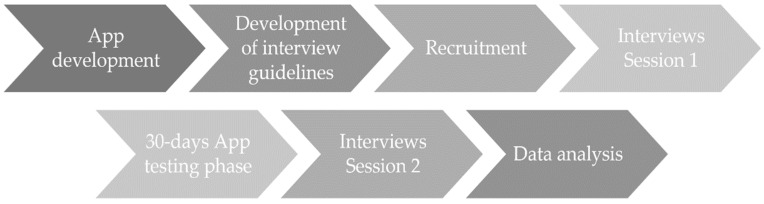
Visual flow of methodology.

Participants were asked about their experiences with suicidality and NSSI as well as their experiences with DBT skills and apps through two semi-structured in-depth interviews per participant. The separate interviews on two different dates enabled us to capture a change in perspective over time, which was achievable using the longitudinal qualitative research approach. This approach provides the possibility to capture experiences of individuals and groups in a more holistic way and allows for in-depth comprehension beyond a cross-sectional point in time [32,33]. A total of 26 interviews were conducted, where 13 participants were interviewed both before and after a 30-day app trial period using separate interview guides. As there was no new information gathered by the time the responses of 13 participants were reviewed, data saturation was accomplished in the course of the first interview phase, which is why recruitment was stopped at that point. The conversations lasted between 30 and 45 min and were subsequently transcribed verbatim. Appendix A Figure A4 visualizes the executed procedure of the study.

Originally, data triangulation was selected as a quality assurance strategy in order to eliminate systematic bias, and generate maximum theoretical insight. With this in mind, both data collections were planned to take place at different locations. The aim of local triangulation is to eliminate thought associations between the interview partners, and is therefore conducted to counteract unconsciously distorted statements [34]. However, due to the ongoing COVID-19 pandemic at the time of the study, the interviews took place online via the Zoom videoconferencing software. The software simultaneously enabled interference-free recording of the interviews and subsequently converted the video recordings into an audio format. A significant advantage of conducting data collection via Zoom was the individual option for participants to turn off the camera during the interview. During the interviews, participants expressed a high level of satisfaction with this option and informed the interviewer that they might not have agreed to a face-to-face meeting.

### 2.4. Interview Guidelines

The interviews primarily focused on participants’ thoughts and feelings regarding suicidality and NSSI. In total, two different guidelines were created, one for the first series of interviews and another one for the second series. The second guide was a slightly modified form of the first version as it contained questions that would have been irrelevant if repeated. The guiding principle used during the modification of the first interview guide was the comparability of the results at a later stage. The two guides designed in the course of the study were based on six relevant topics from the Self-Injurious Thoughts and Behaviors Interview (SITBI). The SITBI is a standardized interview with proven high reliability and validity [35]. The themes on which the semi-structured interview guides were based were suicidal ideation, suicide plans, suicide gestures, suicide attempts, and NSSI. The second semi-structured interview guide was strongly oriented to the first guide. It was partially supplemented or replaced by new questions. Since the two interview series covered sensitive topics, maintaining a high level of safety for the study participants was top priority. In view of this circumstance, the interviewer had to minimize the risk by constantly appraising the interview situation, and by responding with empathy to the emotions and affects that arose during the course of the interviews. If necessary, the interviewees had the option of changing the topic or ending the interview altogether at any time. Based on the experience of the principal investigator in the field of psychiatry and psychotherapy, sensitive situations could be adequately evaluated, and the conduct of the interview thus adapted accordingly. For example, if some participants felt distressed when asked about suicidal ideation and NSSI, questions were rephrased or skipped.

### 2.5. Ethical Considerations

Informed consent was obtained in written and verbal form from participants prior to the first interview session. In order to be able to correctly assign the collected data, participants were pseudonymized. Pseudonymization was carried out by coding names with consecutive numbers, whereby only the study authors were able to draw conclusions about the corresponding persons on the basis of these codes. The analyzed data were tagged with these codes and stored on a private PC with secured access. In case information that allowed third parties to draw conclusions was revealed, this content was selectively blacked out, alienated, or generalized without altering its meaning. Audio files were fully deleted after finalizing the verbatim transcription. This study was approved by the ethics commission of the University of Applied Sciences Campus Vienna (EKNr: 6/2021).

### 2.6. Data Analysis

The interviews were manually transcribed and analyzed using thematic qualitative text analysis (TQTA) according to Kuckartz [36]. TQTA features a multilevel process of categorizing and coding, where at first the data is coded according to major categories derived from the general guidelines used in data collection. Subsequently, these categories are enhanced and differentiated before the dataset is coded again. By means of this constant process of comparing and contrasting, the consecutive category-based analysis gains refinement and explanatory power. Based on the underlying research objective, a deductive category system comprising eight main categories was created. The collected interview material was then coded by assigning text sequences to corresponding categories while the initial category system was inductively adapted, giving rise to six main categories (Table 1). In the context of TQTA, this is referred to as deductive–inductive coding. For the analysis of the follow-up interviews, the final category system was used for coding. Subsequently, an analysis of the content within the sub- and further subcategories resulted in a descriptive comparison between both interview sessions. This deductive–inductive approach is consistent with a recommendation for the analysis of longitudinal qualitative research [32]. Both the transcription and coding process were conducted with the software MAXQDA 2020.

## 3. Results

### 3.1. Experiences with DBT Skills

The results revealed that participants expressed both positive and negative experiences with DBT skills. Each participant had previous experiences with DBT and DBT-A, primarily due to hospital stays and psychotherapy, and their information on DBT skills was limited to two different sources, namely healthcare institutions and online platforms. While most respondents pointed out they were usually taught new DBT skills by healthcare institutions, especially in hospital psychiatric wards, some participants stated they needed alternative ways of reducing tension. These participants reported various ways of seeking relevant information about DBT skills, mainly through online platforms and self-help forums. However, most of these participants conveyed their frustration that many suggestions for self-help were not inspiring enough or unhelpful in dealing with their symptoms.

Several interviewees said they had been taught a list of skills during their inpatient stays. However, they felt that there were no differences in the kind of skills provided by the healthcare institution between the first and second data collection periods before and after the app testing phase. In fact, those interviewed felt that the lists of skills provided by the healthcare institutions were not necessarily useful for them:

*I was given a multi-page list printed out in the hospital of activities to do when you are very tense to get you down. I still have that list, even though I find that it does not necessarily have the most useful things, well, for me personally just not the most useful things, on it. So, not things that I can then apply. Then there are also, for example, things like ironing on it or so. Things like that. Yes, that maybe relieves a bit of tension, but you do not always have that available.*

*(Female, Age 22)*

Nevertheless, acquiring DBT skills in general was a positive experience for participants. One participant emphasized that “*simply the fact to know many skills means to have a certain safety net to fall back on*” (female, age 20). After the app testing phase, participants continued to experience mixed views of the resources available to them. Some of them stated that they already knew enough DBT skills and hence did not see the need to acquire new ones. One participant reported having obtained insufficient skills-support during her inpatient stays at various facilities, which gave her the feeling of receiving inadequate treatment. Although respondents generally accepted and recognized the functionality of DBT skills, they felt that this functionality was linked to certain prior conditions that needed to be met in order to make use of the skills.

Respondents were consistently eager to learn new methods of reducing tension and explicitly wanted to know better ways of dealing with this than to incur self-injury. Despite a certain repertoire of techniques that many participants had already acquired before testing the app, there was a general feeling of inadequacy when it came to applying these skills when they were most needed:

*I don’t feel like I already know enough skills and I should seriously work on that now, but I am hindered by the distraction, a certain indifference that sometimes appears.*

*(Female, Age 21)*

### 3.2. Phenomenon of Self-Harm

Participants described their experiences of self-harm in terms of behaviors associated with NSSI. Compared to the first interviews, NSSI was slightly less frequent during the follow-up. Participants had difficulties in estimating the frequency of NSSI, and attributed this to situational memory lapses or their uncertainty of the definition of self-harming. The methods of self-harm used by respondents in terms of NSSI differed from one another and were strongly influenced by their own individual characteristics. Most respondents mentioned that before the first interview took place they had experienced self-inflicted scratches, superficial cuts or stabs with broken glass or knives, broken bones, burns from cigarettes, and hitting their own body, objects or walls over a period of time. However, when the second interview was conducted, most respondents reported having reduced acute NSSI. Further, most participants did not experience severe incidents during the app testing phase, as they continued to refrain from acutely harming themselves. Nevertheless, some participants expressed other kinds of self-harm in the form of excessive food intake due to craving, and scratching their skin due to high stress levels.

Some of the interviewed persons stated that they suffered constant self-destructive thoughts that varied in intensity depending on the situation or occasion. In particular, they frequently experienced negative thought circles or volatile fluctuations in self-destructive thinking. Consequently, respondents’ perspectives on self-destructive thoughts did not undergo recognizable changes during the course of the study:

*I think that it can change very quickly. Well, not in general, but if something should happen that makes me feel really bad again, then it can fluctuate again very quickly. So I think that would be possible.*

*(Female, Age 19)*

The risk factors and triggers that were reported to have led to self-injury were intrapersonal, interpersonal, or situational. In terms of intrapersonal risk factors, poor self-worth and self-hatred were recurring themes. One participant stated that the shorter the time interval between incidents of NSSI, the more likely NSSI was going to happen. Self-injury was mentioned by some respondents as a measure against incipient or existing dissociations, and said it helped them to stay focused. In fact, several participants referred to NSSI as a pastime. As it turned out, the issue of intrapersonal factors was less addressed during the second data collection. If so, difficulties in this regard were mentioned in connection with drug addiction, flashbacks, and thought circles.

In conjunction with self-harm, high subjective importance was attributed to interpersonal relationships and contacts. Participants specifically referred to relationships with family members, meetings with friends, or exposure to the public as risk factors. Some of the interviewees recounted occasional self-inflicted violence to gain attention from their social environment. During the second interview, only a few respondents commented on interpersonal factors being potential causes of NSSI. However, these problems were consistent with the statements made during the first interview session.

In addition to intrapersonal and interpersonal processes, situational influences were also mentioned as a risk-related factor. Participants reported the use of transport and personal triggers as most stressful. Some participants, particularly in the follow-up interviews, stated that they experienced stress at school and generally felt more unstable due to that:

*Partly, it is simply certain situations. often situations that remind me of some kind of trauma. (…) Situations that trigger my fear of loss are usually a big deal for me.*

*(Female, Age 19)*

### 3.3. Feelings Related to Self-Harm

The feelings related to self-harm that were experienced by interviewees were complex, as they expressed both positive and negative attitudes toward self-injury. Participants compared the effect of NSSI with the intake of psychotropic substances and drew similarities between them. They emphasized the euphoric effects and addictive potential in both. Some of the interviewees spoke of a feeling of relief in connection with self-destructive actions, after a NSSI:

*It is like when you are in a room that has not been aired out in years, and then you open the window and take a really deep breath.*

*(Female, Age 21)*

Although some respondents reported that NSSI was better than any medication, they also claimed that the self-injury was associated with insecurities, and that this needed attention. One participant frequently emphasized her gratitude for having been through the experience of NSSI, as she managed to stop inflicting self-harm and was thrilled to be a role model to support other people with self-harming issues in the future. However, during the follow-up interviews, most participants did not claim that their attitude toward self-injury changed. In fact, one person described self-injury as a communicating mediator between the inner emotional world and the social environment.

On the whole, participants were able to provide concise descriptions of their emotions toward NSSI and suicidal behavior. They mentioned self-hatred, disappointment, anger, sadness, guilt, shame, despair, fear, shock, aggression, tension, craving, doubt, effort, emptiness, and loneliness to describe their emotional experiences with this issue. The interviewees’ perception of this did not change during the follow-up interview, although NSSI was less important compared to the first interview. Besides relating experiences of positively and negatively valenced emotions, some participants also felt emotional indifference toward self-injury and suicidality, and spoke of feelings of apathy or lethargy in this regard:

*During the self-injury, I think there is not much going on emotionally, because then the focus of attention is simply on the action and there is not much room for anything else.*

*(Female, Age 22)*

Respondents reported individual ways of dealing with scars, which originated from past self-injuries. One participant explained that visible scars were not a problem since they could be tattooed over. Other interviewed persons also mentioned using certain clothing as a way of hiding scars, and claimed that they only injured themselves on body parts that were usually not visible to others. During the second interview, several respondents reported feeling a certain emotional distance from self-destructive behaviors and thoughts. However, none of them were able to give any particular reason for this.

### 3.4. Dealing with Disorder-Specific Symptoms

In terms of dealing with disorder-specific symptoms, individuals included in the study had their own perspectives on the selection of DBT skills that were recommended. Participants generally identified positive DBT skills as those that could be used universally as well as in different situations. They also explained that the stimulus intensity of a skill must be adapted to the severity of their distress, in order for it to be effective. Nonetheless, respondents reported that both active and passive variants of DBT skills can be effective, depending on the type of tension. During the interviews, participants emphasized that a well-planned combination of several DBT skills can be an effective and sensible thing to do:

*I do not think there is one good skill, but it is about combining skills as sensibly as possible. The best skills do not help if you are not there for yourself a little bit afterwards.*

*(Female, Age 22)*

Although the interviewees agreed that using physical and cognitive stimuli-based skills were fundamental to counter high distress levels, they found these skills to be only effective for them at low to moderate distress levels. Respondents explained that extreme situations were only manageable with NSSI, taking acute medication, or using skills chains. In addition, respondents emphasized that an important way of dealing with their symptoms was to sensibly combine various skills. During both interviews, participants mentioned the use of a variety of skills which addressed different physical senses. These included gustatory, olfactory, thermal, kinesthetic, and cognitive stimuli. According to some participants, physical activities with pets were also highly important to them, and this was emphasized during the second interview session. They claimed that relating and interacting with their pets was an essential therapeutic resource. Many interviewees referred to mindfulness exercises based on DBT as an effective skill for them. Nevertheless, during the follow-up, one person expressed a rather negative attitude regarding mindfulness skills, stating that these could only be used under very specific conditions and were therefore only effective to a limited extent.

Respondents additionally underscored physical exercise and sports as effective ways of reducing tension, mainly through distraction, exhaustion, and physical exertion. Some participants reported either listening to music or making music themselves. One individual expressed a contrasting opinion on the effect of music, stating that it can both evoke positive thoughts and intensify negatively valenced emotions. The respondent added that the state of arousal as well as the trigger causing it were criteria for the effectiveness of music as a defusing skill. During the second interview series, participants were less focused on physical activities and music, but otherwise reported similar outcomes as they did in the first interview:

*For a while, my therapist gave me chili peppers, so that whenever I simply noticed that I was having bad thoughts, or that I was getting restless or something, I bit into the chili pepper and that helped relatively well.*

*(Female, Age 18)*

The interviewees mentioned that their social environment was an important facilitator when dealing with their symptoms, and explained that this contributed to the development of certain behaviors. They attributed their social environment to potentially influencing them both positively and negatively, and highlighted that families and friends were main factors in this structure:

*First of all, I have my boyfriend as my main anchor, I would say. The thing is, I live with him, so to speak, and he’s known me since the time when it was really bad and therefore knows how he should deal with me in these situations, how he can bring me down.*

*(Female, Age 18)*

Additionally, participants mentioned having contact with healthcare institutions or psychotherapists as essential. In this regard, they described friendships from previous hospitalizations as important relationships as these were based on emotional connections and common experiences. Moreover, one respondent mentioned experiencing positive attention from the opposite sex, which was a feeling that partly strengthened her self-worth. Some participants reported using computer-based tools when feeling highly distressed. Respondents stated that video games and music production were helpful in calming them down when feeling highly distressed. A few respondents cited online chats and forums as important resources, while others mentioned the use of mobile devices in general, as well as certain apps as effective tools to combat NSSI and suicidal behavior.

Participants in general also described psychotropic substances as very effective in terms of dealing with acute situations. Among these, they found nicotine, alcohol, and cannabis to be most alleviating of their symptoms and hence consumed them frequently. Further, respondents described the use of emergency medication as particularly effective in relieving them of high distress, and referred to the primary use of benzodiazepines, a group of drugs with anxiolytic and sedative effects, and low-potency antipsychotics, also causing sedation. Respondents also made statements about their regular medication, where antidepressants and antipsychotics were mentioned most frequently. Some participants stated that they sometimes combined their prescribed medication with illegal drugs:

*It is true that in the long run, marijuana aggravates depression or anxiety disorders. I have even noticed that in myself. But in combination with the medication it works quite well. Maybe I’m risking a bit much right now, but as long as it works, it’s good.*

*(Female, Age 21)*

Participants referred to several limitations they were confronted with when they wanted to use DBT skills. According to one person, a blindness to DBT skills arose during high states of arousal, which made it impossible to apply them adequately. Another interviewee said that the use of skills as a substitute for NSSI would be easier if she was not at home, while another person commented that DBT skills felt more like an oppression than an adequate compromise to avoid self-injury. Some respondents stated that suggestions from healthcare professionals regarding general skills were often insufficient, as skills could only be effective if they were personalized. One person said that some skills only had a stress-reducing effect when experiencing a certain level of tension, beyond which the potential to inflict self-injuries was raised. Two participants agreed that during acute situations, personal difficulties hindered them from asking for help, while two other participants mentioned the environment as a possible limitation, as certain skills could not be used in public places. With regards to this, respondents mentioned their unwillingness to avoid self-injury, their inability to recall learned skills from memory in stressful situations, their desire not to disturb the social environment and therefore not ask for help, and their unwillingness to look for certain skill objects if they were not immediately available.

### 3.5. Prevention of Self-Harm

The majority of participants reported that the main reason they avoided NSSI was to prevent long-term physical damage. For instance, respondents pointed out having suppressed their compulsive NSSI tendencies many times in order to avoid creating visible scars. During the second series of interviews, one interviewee commented on the fact that the last self-injury occurred some time ago and that she had “*realized that it just does not do as much good as it used to, or that there are just different strategies now to avoid it and there is no urge any more*” (female, age 19). One person expressed that avoiding NSSI was part of her self-responsibility, and found it essential for her personal development and growth. Another participant disclosed that cutting was associated with effort, thus not suitable as a short-term coping method:

*So the cutting was just really exhausting, because it made a mess and the wounds did not heal and then you had to kind of put bandages and something over it and it kept coming open. I like to have things just for the moment and not deal with them afterwards and that was just exhausting. Apart from destroying my skin.*

*(Female, Age 21)*

As many participants stated, an essential influence on NSSI was social perception, and that they did not want to disappoint closely related individuals. Moreover, people in their social environment were considered as great resources for dealing with BPD symptoms. Participants talked about experiencing shame when scars were visible from outside. During the follow-up interviews, one person stated that she did not want strangers to have bad impressions of her due to visible scars. Further, respondents said that regular contact with a peer group helped them find effective coping methods. Some interviewed persons also reported that having a pet was a valuable resource to them, and that taking responsibility for an animal contributed to them maintaining a better sense of self. In the course of the second interview session, one interviewee vividly summarized the general view of persons suffering from BPD on people in their social environment:

*The motivations have remained the same as when we first spoke. That this is something I do not want to do to other people, because I know that it is not easy to have a contact with a, as stupid as it sounds, “borderliner” or a self-harming person. That this is not nice for other people, I know that. And accordingly, I do not want to do that to anyone around me. That they worry about me and that they constantly worry and check if I have not done anything or whatever. I just do not want that. So that has actually remained the same.*

*(Female, Age 22)*

### 3.6. Attitude toward Skills Apps

In general, respondents had positive opinions about the use of mobile apps in terms of emotion regulation. For instance, participants emphasized the permanent availability of their mobile device, and the array of possibilities offered by the DBT skills content in particular. Although attitudes toward a skills-providing app were generally positive, some interviewees also expressed concerns about the functionality of the app and the potential difficulties in providing help in an interpersonal way:

*I do believe that in some cases an app can help really well with tension, but I actually believe that it is difficult to program an app in such a way or to make it so user-friendly in a way that when you are in a high level of tension that you can actually experience the app as helpful. (…) There can certainly be situations where an app is sufficient. But I think that the interpersonal aspect gets lost. For me, one of my best skills is talking to someone.*

*(Female, Age 21)*

While some persons could imagine browsing the contents of the app and trying out unknown skills, some of them pointed out that the use of a mobile app could possibly be more effective for acute emergencies than for long-term skills training. During the interviews after the 30-day app testing phase, the interviewees mentioned a variety of points which they considered necessary for the proper functionality of a mobile app. For example, these included uncomplicated usability, appealing content structure, modern design, interesting descriptions, clarity, multimedia content, occasional app notifications, explanations of DBT basics and skills training, integration of a collection of the most important skills, choice option on types of skills, easy-to-understand information, integrated mini-games, integration of gamification elements, applicability in acute situations, the mobile app as a companion to a real therapy or skills group, a chat function for group members when used within a skills group, and the integration of skill chains that could be worked through by users. One participant reported that the high information content of the investigated app might be more suitable for an audience inexperienced with DBT since more experienced persons might already be familiar with most of the content. Referring to this, respondent suggested integrating an option to adapt the provided content based on individual experience.

Table 2 provides an overview of the participants’ key characteristics and their main ideas for a successful DBT app.

## 4. Discussion

The objective of this study was to investigate the attitudes of transitional age youth (TAY) with borderline personality disorder (BPD) toward using a dialectical behavior therapy (DBT)-based mobile app. Generally, participants showed a high level of acceptance regarding the use of the investigated mobile app. Despite little change in perceptions of suicidality and NSSI, respondents were able to envision the utility of the app, using it as a supportive measure for in-person psychotherapy sessions. It should be pointed out in this context that affected persons choose the type of self-inflicted violence according to their own individual criteria, and that personal needs and compulsions are in the foreground [37]. For a DBT skill to be fully effective, it is important that it produces a similar release of tension as does the self-inflicted harm. For this reason, profound engagement with a variety of skills is a fundamental component of DBT [38]. In this study, the smartphone app investigated was hoped to be a promising complementary tool for in-depth personal engagement. However, this effect did not occur during the 30-day testing period. Although participants reported a certain recognition of the possibility of using the app as a permanently accessible source of knowledge, the offer did not bring about any noticeable changes in the subjective view of suicidality and NSSI. However, participants were receptive to the app as a tool for acquiring knowledge. To a large extent, this could be related to a strong understanding of the disorder that most participants had, and their active desire to change their general life situation. People affected by BPD see themselves exposed to extreme psychological strain, which is characteristic of the mental disorder [39]. For this reason, respondents expressed a high interest in the app as a method for knowledge transfer as well as a high willingness to learn new ways of dealing with issues related to the disorder. Nevertheless, it must be mentioned at this point that the willingness to learn that was revealed within the first series of interviews mostly diverged from the actual effort carried out with regard to the app use within the test phase. It should be noted that the willingness of app users to engage in an app lies in how appealing it is to them [40,41]. In this context, the mobile application offered too little stimulation to achieve an active engagement hampered by the users’ dysfunctional behavior and associated mental processes. During the month of testing a slight reduction of suicidal ideation and NSSI tendencies occurred, although participants related this change to different extrinsic factors and not to the DBT app per se. In comparison, other DBT apps were able to achieve more positive outcomes in individuals with BPD [14,24]. However, these studies did not focus on TAY, but adults in general, which is why this difference may be owed to the difference in impulse control, as adults with BPD often show more control and stability [42]. This could be due to higher levels of life experience and more time to learn the needed skills to cope among adults.

An essential point to consider with regard to suicidality and NSSI are the associated risk factors and triggers [43]. As can be seen from the results, a breakdown of the general risk factors and triggers into intrapersonal, interpersonal, and situational influences took place within the study. It should, however, be noted that these influences interact with each other on a continuum [44,45,46]. All factors that affect an individual are primarily processed intrapersonally, and are hence independent of whether the triggering factors are of intrinsic or extrinsic nature [47]. The majority of risk factors and triggers elicited after the app testing phase were decidedly referred to as situational. In this context, it is important to mention that the upcoming school leaving examinations and the high intensity of exams due to the approaching end of school year significantly influenced general well-being of some participants. The occurrence of NSSI and suicidal ideation are particularly pronounced in individuals with increased vulnerability to BPD. Stress has a high impact on this [39].

Maladaptive behavior patterns in interpersonal relationships are a typical symptom of BPD. These relationships have a particularly influential role in triggering self-destructive actions [48]. In the present study, this was seen to be the fundamental source of inciting NSSI among participants. Interpersonal communication is highly recommended for TAY with BPD and is characterized by immense complexity [49]. This complexity was also reflected in the course of this study. From a subjective point of view, the interviewed persons ascribed great importance to interpersonal contacts, although it should also be noted that the degree of reflectivity of the selected participants was generally high. Within the interviews, participants expressed an understanding of the relevance of external influences by other persons and were able to articulate this accordingly. Specifically, participants mentioned that they were highly emotionally influenced by external contacts and that this susceptibility caused instability in their affect regulation. That there is an increased risk for evoking NSSI in emotionally volatile relationships has been previously shown [50]. In the course of the two interview series, no pronounced differences emerged with regard to the intensity of interpersonal influence, implying that using the app did not result in any changes in this aspect. However, it should be noted that under other circumstances, interpersonal contacts could also have a positive influence and hence actively prevent self-destructive behavior. This was highlighted by interviewees who expressed their aversion to visible wounds and other aspects that related to affecting their social environment. This aversion remained during the second data collection as well, hence representing an essential factor in preventing dysfunctional behavior.

On the whole it should be noted that participants underwent a relatively limited transformation of their subjective perception regarding NSSI and suicidality. With regard to personal perception, participants showed indistinct changes after the intervention considering the aforementioned phenomena. Suicidal ideation and NSSI had already been issues for the subjects for several years at the time of the study, possibly rendering the influence of a 30-day app intervention to be negligible. Although a few respondents realized the harmful impact of their dysfunctional behavior, the majority of participants expressed an emotional indifference to this kind of behavior. This indifference or emotional passivity may indicate a resignation among individuals affected by BPD that develops over time and is likely associated with individual experiences.

### 4.1. Limitations and Strengths

This study incorporated several innovations in the field of BPD research. Firstly, it was conducted within the natural environment of the included persons. Secondly, a DBT-based mobile app was investigated among a sample of transitional age youth living with borderline personality disorder. Finally, the longitudinal qualitative design offered the possibility to do in-depth interviews and assess participants’ opinions and emotions during the course of the research. This approach allowed a holistic insight regarding the impact of the intervention.

The fact that some participants were in the preparation phase for their school leaving examination during the app testing period could have been a limitation. This may have resulted in only little attention being paid to the active testing of the application. In view of this, it could also be assumed that the small amount of time spent using the app had a substantial impact upon the data collected. Moreover, there was an imbalanced distribution of gender, with 92.3% of participants being female. Additionally, the duration of the app testing phase of 30 days may have been too short. This restriction resulted from the limited time resources available for the implementation of this study. An extended testing period could possibly yield different results. Moreover, a longer-term testing of the application is required in order to do justice to a holistic approach. In connection to limitations concerning the intervention, it should be noted that using the mobile app without strict guidelines from the study management may have resulted in the software not being actively used. A modified approach could bring about different results in this regard. Yet, the focus was on promoting the autonomy of participants, which is why a predefined schedule would not have been target-aimed in terms of self-reliance. Finally, in the context of the interview situation, there were several limitations and strengths due to the ongoing COVID-19 pandemic during the period of the study. The primary methodological consideration for this research was based on data triangulation according to Flick, which was opted for as a preventive measure against systematic bias [34]. In this methodology, the two interview series would each have been scheduled at different locations. However, in order to comply with pandemic-related contact restrictions, no face-to-face meetings with the subjects were possible, and hence a web-based videoconferencing service was used. Conducting the interviews in face-to-face meetings may have potentially led to different results.

However, it should be noted that having the interviews in an online format certainly brought advantages that were highly valued by some interviewees. Participants felt that the possibility of ending the interview at any time gave them a certain sense of safety, which may have contributed to more open conversations. Further strengths of the study were the familiarity between the principal investigator and most participants, as well as their high willingness to contribute to the research.

### 4.2. Future Outlook

These insights gained should be used to further develop a DBT-specific mobile application. Such an app should be expanded by the functions and elements addressed by our participants, primarily by adding multimedia content, such as video, audio, and other formats. Certification of an app as a medical product would create the basis for the implementation of a variety of diverse features that could provide more in-depth experiences for users. In concrete terms, this could allow for functions that require input from users, such as a digital mood diary, to be embedded. Since a qualitative research approach was used in this study, future studies with a quantitative orientation could contribute to a more holistic understanding of the phenomena investigated. For instance, the correlation between the occurrence of NSSI and time since the last NSSI could be considered as an objective for additional quantitative research, allowing for a mixed-methods approach that could prove promising for this purpose. The time since patients were discharged from the hospital and their emotional stability when utilizing a DBT app may be a matter of interest for future studies. Given that the focus of the study was the experience of having a companion app on hand, no data on the frequency of the use of the app was gathered. Nevertheless, in order to accurately examine the effects of the app as an intervention, further work should quantify its use. For instance, use statistics may be captured, or subjects could be given specific guidance on the time period, duration, and type of usage required. In this way, it would be possible to derive optimal conditions of the app usage. On the other hand, it must be considered that such a guideline could affect the motivational level of participants. Further investigations should also be made on specific parameters such as having a longer testing period and a more balanced gender distribution. In future studies, possible implications for practice should also be explored. One way is to investigate how the DBT app could function as a companion to personal therapy. For example, patients would be given the opportunity to repeat and practice the content learned in individual or group therapy upon individual request and at one’s individual pace. The therapeutic process could benefit considerably from this and have a motivating effect on the users.

## 5. Conclusions

In summary, it can be stated that the provision of an app that integrates dialectical behavior therapy (DBT)-specific content has met with a positive response from the group of transitional age youth (TAY) with borderline personality disorder (BPD). The patients were willing to integrate such a system as a supportive measure in their everyday life, but needed to tie this willingness to specific conditions related to the functional scope of the application. Respondents preferred a smartphone app that focused on acute situations, was easy and intuitive to use in an emergency, and at the same time provided concrete instructions for action. With regards to its long-term use, respondents were not interested in the sole dependence on autonomous software for smartphones, but preferred to use it as an accompanying intervention to psychotherapy in an individual or group setting. It should, however, be noted that the influence of the 30-day app testing period only marginally reflected the views of respondents in terms of suicidality and NSSI. Furthermore, these findings may only be useful to a limited extent for individuals between 15 and 26 years with borderline personality disorder, since the sample size and gender imbalance in this study does not allow for generalizations. Furthermore, this exploratory study did not aim to investigate the effect of DBT. On the basis of the findings obtained, the integration of a DBT app into the everyday life of TAY with BPD is possibly beneficial to their subjective feelings, but this effect has yet to be confirmed through further research.

## Figures and Tables

**Table 1:** Final category system.

Main Category	Subcategory	Further Subcategory
Experiences with DBT skills	Skills transfer	Individuals framework
		Institutional framework
	Personalized skills	Availability
		Attitude
	Willingness to learn	
Phenomenon of self-harm	Self-destructive behavior	Frequency
		Types of self-harm
	Self-destructive thoughts	
	Risk factors and triggers	Intrapersonal
		Interpersonal
		Situational
Feelings related to self-harm	Positive emotions	
	Negative emotions	
	Emotional indifference	
Dealing with disorder-specific symptoms	Skills selection criteria	
	Applied skills	Physical and cognitive stimuli
		Mindfulness
		Exercise and sports
		Music
	Social environment	
	Psychotropic drugs and intoxicants	
	Limitations	
	Technology in the context of emotion regulation	Computer applications
		Streaming media
		Mobile apps
		Websites
Prevention of self-harm	Intrinsic factors	
	Extrinsic factors	
Attitude toward skills apps		

**Table 2:** Transitional age youth (TAY) participants with borderline personality disorder (BPD), based on sex, age, education level, current occupation, time since discharge from hospital, occurrence of NSSI during the app testing month, and ideas for an effective DBT-based app.

Participant Code	Sex	Age	Education (ISCED- 2011-Level)	Current Occupation	Time since Discharge from Hospital	NSSI during App Testing Month	Main Ideas for a DBT App
01	Female	20	5	University student	1 month	None	usability; modern design; simple descriptions of DBT skills; multimedia; focus on emergencies
02	Female	19	2	High school student (imminent school leaving exam)	1 month	None	occasional notifications; information about DBT skills training; various skills domains
03	Female	19	5	High school student (imminent school leaving exam)	8 months	Yes	focus on emergencies; personalized skills training
04	Female	21	4	University student	11 months	Yes	app use as a supportive measure in personal psychotherapy
05	Female	18	2	High school student (imminent school leaving exam)	12 months	None	focus on emergencies; quizzes; personalized DBT content
06	Female	19	2	Unoccupied	1 month	None	integrated mini games; simple skills descriptions
07	Female	21	3	Unoccupied	10 months	None	personalization of DBT skills; personal organization of skills trainings; accessibility
08	Female	20	3	Unoccupied	<1 month	None	focus on emergencies; skills suggestions based on user input
09	Female	22	3	Unoccupied (recent completion of school leaving exam)	6 months	None	focus on emergencies; clear instructions for DBT skills trainings
10	Male	19	3	University student	2 months	None	gamification; achievements; additional to personal psychotherapy
11	Female	18	2	High school student (imminent school leaving exam)	9 months	Yes	notifications for exercises; achievements; focus on mindfulness
12	Female	22	5	University student	6 months	None	mood diary; sticky notes
13	Female	23	5	University student	<1 month	None	gamification; focus on long-term skills training; notifications

## Data Availability

Limited dataset is available from the corresponding author upon reasonable request.
